# Insights from the culinary arts for medical educators

**DOI:** 10.15694/mep.2017.000010

**Published:** 2017-01-18

**Authors:** Poh Sun Goh, John Sandars

**Affiliations:** 1 National University of SIngapore; 2 University of Sheffield

**Keywords:** eLearning, Technology enhanced learning, Culinary arts, Medical education

## Abstract

This article was migrated. The article was marked as recommended.

## Letter

Dear Editor,

There has recently been a phenomenal global interest in reality television shows related to the culinary arts. In these shows, celebrity chefs and amateur cooks describe and justify how they prepare and deliver a variety of meals. We feel that there are close parallels between the culinary arts with the teaching and learning practices of medical educators, and this culinary analogy can be used as a means to help medical educators think about their educational practice, and to develop their scholarship in medical education.

The aim of a good chef is to not only provide the essential dietary nourishment but also to engage the senses of their diners so that the meal is satisfying and memorable. To do this effectively requires training, experience and an experimental mindset in the chef, as well as an awareness of the tastes, food preference and palate of their diners. The chef works with raw ingredients, cooking utensils and methods, as well as follows a range of cooking recipes, to produce, and reproduce different dishes. There is a need to have a well stocked larder, to source the freshest and best ingredients, to partly cook and prepare some of these ingredients in order to be able to efficiently deliver multiple orders on demand. The best chefs continually seek to improve their recipes, finding new ingredients, and cooking processes, as well as develop innovative cooking methods; while continually seeking feedback from peers and patrons. 

A good teacher likewise aims to facilitate learning, and provide an engaging learning experience for students, as an engaged student is more attentive, and will spend more time and effort to learn. As teachers we also aim to produce consistently good teaching, to satisfy the intellectual and training needs of our students. To be a competent chef requires training and experience; and we all acknowledge the necessity of training, both formal and informal, for clinical practice. Similarly, faculty development, and training in educational pedagogy and good practice supports and augments our teaching. Chefs improve by spending more time in the kitchen, by engaging in the practice of cooking. We improve as teachers the more often we teach, particularly if we reflect upon our practice, and continue to seek feedback. 

Improving our teaching also requires us to refine our teaching, experiment and innovate with different and new instructional formats and paradigms, with a scholarly mindset and engage in the cycle of scholarship; with the process of dissemination, peer review and academic discourse helping us to reflect upon, refine, and improve our own teaching, as well as contribute to the dissemination of useful instructional practices for other educators.The very best chefs challenge themselves on a regular basis to change and improve their recipes and menus, to travel and be inspired by other chefs, and to visit and work with other excellent chefs in their kitchens. These chefs seek to share their recipes, to teach novice chefs, and engage with other chefs to discuss and share ideas of how to improve their culinary practice. As teachers, engaging in faculty development to share our experience with and teach other educators; and participate in educational scholarship both informs our own educational practice, and also improves it. The process of documenting, systematically evaluating, modifying and refining the components of a cooking recipe, and the cooking methods, in order to produce a consistent dish, is very similar to the process of action research in medical education, where each component of an educational intervention is examined in relation to each other, within the implementation context, in collaboration with the learner. The science of cooking and the science of educational practice are similar.

To be effective as educators requires a keen awareness of the learning needs of our students, as well as their learning preferences. To prepare our instructional program requires us to assemble useful instructional material, which nowadays is in digital form, as text, illustrations, animations, multimedia and videos; also increasingly interactive scenarios and in the future digital overlays. We will design and create some of these learning resources, but the majority of these will increasingly come from online digital collections and repositories, both in house and open source online collections. Focusing our efforts, both individual and collectively, to curate and index not only effective learning resources online, but also individual components of these resources as "reusable learning objects", will allow us to improve the efficiency with which we assemble and refine our customised teaching programs. This approach is very similar to how an experienced and trained chef will source for ingredients to prepare a wide variety of cooked dishes "on demand". The great advantage of digital educational educational material, compared with non-digital formats, or tangible cooking ingredients, is that only one copy of each reusable digital object is required, and this copy can be used, reused, and recombined as many times as required by a teacher, as these digital learning objects never lose their freshness or become "used up"; and there is no limitation on the same copy being used by many other teachers.

A concerted effort to collect and index online these reusable pieces of text, illustrations, tables, references, video snippets, and interactive elements; with appropriate citation, and permission to use and disseminate this material with acknowledgement of the source of this material and correct attribution is one compelling future that more of us could collectively work toward at both a local, and international level. We can further refine the usefulness of this digital repository, by creating partly finished combinations of digital learning material, and make these also available in the online repository. This is very similar to how having access to partly prepared cooking ingredients, which can be quickly reheated, allows a chef to efficiently and more quickly prepare consistently good meals in a busy kitchen. 

A good teacher is continuously observant of student learning behaviour, and seeks evidence of student engagement with the learning material and experience, as well as evidence of learning, and also proactively solicits feedback from students and peers to continually improve the learning experience. Good chefs can be seen circulating amongst the diners, observing their eating behaviour, chatting with and seeking feedback directly from diners, as well as inviting fellow chefs to sample their culinary offerings. As teachers we have an opportunity to do this both in traditional classrooms, as well as online settings, through our own direct observations, as well as with online behavioural and data analytics. Chefs often modify their cooking, and refine this in "real time". Similarly teachers can modify their teaching, and the learning experience dynamically, in response to the observed behaviour and feedback from learners and peers.

It is useful to take a moment to examine the culinary experience from the perspective and viewpoint of a potential diner. The experience of the diner can be continually increased by exposure to an increasing variety of culinary practices, through travel to different destinations, watching or reading a range of different media and by being offered an increasing variety of culinary practices, from pre-prepared food options available in supermarkets, home delivery to fast food and pop up food outlets, to ever increasing formal dining options. With the internet, and mobile technology; an enormous breadth, range and depth of educational and learning material is now available "on demand" at the fingertips of an increasingly number of students worldwide. There is now an opportunity for students to not only look up something, but also to increasingly select a customised learning and training programme for themselves; ideally built in consultation with an experienced teacher.

The challenge for novice and beginning students is to decide what is important, and what the best sequence of learning experiences is ideal for their stage of training. Our goal as educators would ideally be the delivery of a personalised, adaptive learning and training experience. This is the role of a personal tutor or instructor; similar to the role of a personal chef. For novices, there is a role for a set menu. While for intermediate and advanced learners, or diners, there is an increasing role for an a la carte dining or learning experience, or presentation of a buffet of dining or learning options. Just as novice diners have little prior experience, and need more guidance; novice learners have little prior knowledge, and require not only more guidance, but also a structured learning experience. Intermediate or advanced students, or experienced diners however have a better idea of the learning, or dining options available, and are in a better position to select and combine dishes, or sequence learning activities for themselves, and require less guidance from a teacher, or culinary expert. 

Ultimately we should aim to train our students to learn themselves, to evaluate their training needs, and the quality of both instructional materials and the instructional experience. This is similar to training diners to cook and feed themselves; or how to find the most nutritious and tasty food. The food preparation and cooking process can be made visible and transparent in open kitchens, just as the actual dishes available on a menu can be made visible. The aim of a culinary academy is to train student chefs, with the cooking products of these students available for inspection and review. Similarly, the medical education process, its components, and its outcomes can be made visible for inspection, assessment and evaluation. Our students should not only "know, and know how", but be able to "show how, and do", as medical practitioners. 

We hope this exploration of several analogies between cooking and dining, compared with the teaching and learning experience, will stimulate medical educators to take advantage of each visit to a grocer or supermarket, and each mealtime as an opportunity to reflect upon, and think deeper, about not only their cooking and dining experience, but also the teaching and learning experiences that they have provided. We hope this process leads to continued refinement, and innovation in educational practice.

## Notes On Contributors

Poh Sun Goh, MBBS, FRCR, FAMS, MHPE, is an Associate Professor and Senior Consultant Radiologist at the Yong Loo Lin School of Medicine, National University of Singapore, and National University Hospital, Singapore. He is a graduate of the Maastricht MHPE programme, and current member of the AMEE eLearning committee.

John Sandars, MBChB, MD, MSc, MRCP, MRCGP, FAcadEd, is Honorary Professor, Academic Unit of Medical Education at the University of Sheffield. He has a major interest in developing the scholarship of medical educators and is a current member of the AMEE eLearning committee. 

## Bibliography/References

## Declarations

The author has declared that there are no conflicts of interest.

