# Supplementary material for: Insights from the culinary arts for medical educators
Source: MedEdPublish (2016). 2017 Jan 18;6:10. [Version 1] doi: 10.15694/mep.2017.000010 (PMC10885217; doi:10.15694/mep.2017.000010)
Supplement: Peer review history

# Reviewer report: Reviewer response for version 1

**DOI:** 10.21956/mep.19426.r28564

**Published:** 2019-03-28

**Authors:** Ken Masters

**Affiliations:** 1 Sultan Qaboos University

This review has been migrated. The reviewer awarded 4 stars out of 5 I enjoyed reading the article, and the analogy seems to fit nicely. I do wish though, that the authors had concentrated a little more on the insights and what we as medical educators can learn from the analogy. There are times that they get into it, and begin to pursue it, but then fall into mainly describing the similarities between the two fields, rather than focussing on what we can learn from the other.Still, an enjoyable read, covering some of the important things that good educators need to be able to do.

Reviewer Expertise:

NA

No decision status is available

# Reviewer report: Reviewer response for version 1

**DOI:** 10.21956/mep.19426.r28562

**Published:** 2018-05-02

**Authors:** Anthony David.M

**Affiliations:** 1 Soho

This review has been migrated. The reviewer awarded 4 stars out of 5 An excellent taste of a letter! Enjoyed the flavour of this dish! The writers comments are very much true and as a medical educator of two decades experience I have seen myself evolve as a better chef from time to time. The analogy seems perfect the T/L experiences we make the learners go through are the delicacies we create.The fact is that no dish can taste the same when made again. The taste and the feedback depends on various factors. Our preparation and delivery of classes or sessions again depend on so many factors. As the saying goes, the proof of the pudding is in the eating. So taking a feedback at the end of every T/L session would be ideal if we have to improve our teaching skills. What I suggest is that we audio/video tape our performances and have a look/hear at it later. This can be a do-able feedback and often helps us see what fools we were!Thank you authors for this delectable letter!

Reviewer Expertise:

NA

No decision status is available

# Reviewer report: Reviewer response for version 1

**DOI:** 10.21956/mep.19426.r28563

**Published:** 2017-01-18

**Authors:** Trevor Gibbs

**Affiliations:** 1 AMEE

This review has been migrated. The reviewer awarded 5 stars out of 5 I enjoyed reading this novel approach to understanding the importance of medical education, I felt that the analogies were "spot on" and the real message was there to see.As someone who recently visited a new restaurant, I would add two other points. At this restaurant, recently opened by one of the winners from the UK TV series of Masterchef, the food was excellent but the service was appalling ( I will not waste time in listing all the problems) and completely ruined the meal and subsequently the evening- pointing to the importance of the staff (or faculty in our analogy) fully understanding and being involved in development of ALL related to and in delivering the goods - there is no point in being innovative if the innovation cannot be understood and delivered well by everyone.Two days later I received through an email a request for feedback, to which I duly replied, stating why I had not enjoyed the meal. To date I have not received any feedback from the restaurant and one can only presume that they take notice of only positive feedback- another strong analogy to medical education. If we are to involve students we have to fully involve students, respect their feedback and take note of the negative effects as well as the positives.The chances are that I will not return to that restaurant, despite the food being excellently cooked!

Reviewer Expertise:

NA

No decision status is available
